# Editorial: Kinin 2022 Meeting, Annecy, France

**DOI:** 10.3390/jcm12093276

**Published:** 2023-05-04

**Authors:** Alvin H. Schmaier, Arije Ghannam, Christian Drouet

**Affiliations:** 1 The Division of Hematology and Cell Therapy, University Hospitals Cleveland Medical Center, Case Western Reserve University, 2103 Cornell Road, WRB 2—130, Cleveland, OH 44106-7824, USA; 2 KininX SAS, 38000 Grenoble, France; arije.ghannam@gmail.com; 3 Institut Cochin, Université Paris Cité, INSERM UMR1016, 75006 Paris, France; christian.drouet@inserm.fr

The Kinin 2022 meeting took place at the Imperial Palace, Annecy, France, from 5–8 June 2022. The opening presentation by Professor Diana Karpman of Lund University focused on the interactions of the renin–angiotensin (RAS), kallikrein–kinin (KKS), and complement systems in inflammation [1]. She presented new data that bradykinin B1 receptor (B1R) antagonists reduced C5a and C5b-9 in cell lysates treated with kaolin-activated normal serum. Both C1 inhibitor (C1INH) and a B1R antagonist reduced C3 fluorescence on renal glomeruli in combined B1R/bradykinin B2 receptor (B2R) knockout mice, suggesting reduced complement activation on glomerular endothelial cells [1].

## 1. Day 1 of Conference: KKS Biology

Coen Maas, from the University Medical Center Utrecht, focused on the mechanism of internal regulation of FXII to prevent premature activation [2]. FXII has a closed conformation to maintain zymogen quiescence and prevent unprovoked BK formation. The fibronectin type II (FN2) domain is an internal regulator preventing FXII from activation by plasma kallikrein (PKa). The EGF1 domain of factor XII (FXII) is an artificial surface binding site. Binding of FXII to the charged surfaces makes it susceptible to PKa cleavage because it “opens-up” the protein. The AlphaFold modeling (https://alphafold.ebi.ac.uk/, accessed on 9 April 2023) suggested that folded zymogen FXII is held together by cysteines. Once reduced or, in the case of the W268R, a variant FXII, the protein is more susceptible to cleavage and activation by PKa and plasmin, respectively [3].

Professor Francois Alhenc-Gelas of Paris-Cité and Sorbonne Université examined the effects of genetic variations in ACE/kininase II in cardiovascular or renal disease on life expectancy in diabetes [4]. The ACE/kininase II gene is a prognosis factor for nephropathy in type 1 or 2 diabetes. Genetic variation in ACE levels was associated with mortality or long-term survival in cohorts of patients with type 1 diabetes. It is the first genetic factor identified as influencing life expectancy in diabetic patients [5].

Bjoern Burckhardt of Henrich Heine Universität, Düsseldorf, described research on the development of next-generation BK assay using LCMSMS. The critical part was the inhibition of ex vivo bradykinin (BK) and related peptides’ generation and degradation during sample collection [5]. This cocktail of inhibitors included sodium citrate, EDTA, hexadimethrine bromide, nefamostat mesylate, 1% formic acid, omapatrilat, and chloroquine. It allowed stable measurement of seven kinin-related peptides, including BK, BK1-8, BK1-7, BK1-5, BK2-9, kallidin, and kallidin1-9. The results suggest that all previous studies on BK levels in disease states need to be reconducted with this methodology.

Bertrand Favier of the Université Grenoble Alpes presented data on minimal reconstitution of KKS to examine its enzymatic model of activation [6]. He examined FXII and KKS activation at 0 °C and at 37 °C. PKa and activated FXII (FXIIa) were active at 0 °C, with *kcat* and *K_M_* values being about five times lower at 0 °C, but the catalytic efficiency (*kcat/K_M_*) was the same. At 0 °C, the cleavage rate of HK was only about 5 min slower that at 37 °C. These results demonstrate that KKS zymogen activation deviates from a Michaelis–Menten mechanism by a faster initial rate and a slower late rate [7].

Professor David Gailani of Vanderbilt University presented mechanisms underlying hereditary angioedema (HAE) in patients with normal C1INH levels [7]. The heavy chain of FXII is involved in surface binding, but, when not surface bound, it restricts FXII activation. The EGF-1 region is the site necessary for surface-dependent FXII autoactivation and PK activation by FXIIa. HK and FXII’s FN2 region limit FXII activation in the absence of a surface. FXII’s FN2 region also binds to negatively charged surfaces to participate in FXII’s unfolding and activation. Further polymorphisms in plasminogen influence BK liberation from kininogens. BK generation from human high-molecular-weight kininogen (HK) or low-molecular-weight kininogen (LK) occurs better with plasminogen Glu^311^ rather than plasminogen Lys^311^. However, this observation was not the same in mice.

Professor Michael Bader from the Max Delbrück Center for Molecular Medicine, Berlin, characterized the function of BK receptors in transgenic animal models. Transgenic rats that overexpressed B2R [TGR(Tie2B2)] manifested with increased fluid and tissue edema and had spontaneous gut angioedema. Aortic rings from transgenic rats expressing B1R [TGR(Tie2B1)] hyperpolarized to both LPS and *des*-Arg^9^-BK, whereas aortic rings from normal rats just responded to LPS. These results show that models of overexpression of vascular BK receptors have biological responses in the absence of any pathological stimuli [8].

Kinga Borsodi from Semmelweis University in Budapest, Hungary, showed that bladder contraction was mediated primarily via B2R [9]. In both human and murine bladders, contractions were independent of acetylcholine, ATP, or prostanoid mediators. The B2R antagonist HOE140 diminished contractions, whereas B1R antagonism had no effect. Stimulation of B2R, but not B1R, initiated bladder contractions. Such contractions were mediated in part by both Gαq/11 and Gα12/13 since mice deleted of these G-coupled proteins had diminished responses.

Elvire Vaucher from the University of Montreal showed that BK is a potential therapeutic target in age-related macular degeneration (AMD) [10]. Both anti-VEGF and B1R antagonism reduced AMD. A B1R antagonist, R-954, which is a non-peptide mimetic, reduced inflammation and neovascularization in a rat AMD model. Laser-induced injury treated with eye drops of R-954, a B1R antagonist, significantly inhibited retinal changes and prevented upregulation of B1R, TNF-α, IL-1β, COX-2, and ICAM-1. This treatment also decreased leukocyte adhesion and retinal permeability. Last, B1R became overexpressed in human eyes with AMD retina, and B1R antagonism reduced retinal inflammation.

Professor Alexander Faussner of the Ludwig Maximilian University of Munich examined re-sensitization of B2R by ACE inhibitors. In receptor transfected HEK293 cells after the induction of ACE, captopril elicited a second Ca^2+^ peak following BK use to induce an initial peak. The addition of the ACE inhibitor strongly increased local BK concentration, allowing for a second stimulation of B2R [11].

Michel Raguet, President of the French HAE patient association, presented an overview of the typical quality of life of HAE patients. These patients who have frequent attacks have a poor-quality social life (in school, work, personal, and family domains). However, Mr. Raguet presented an optimistic future view for HAE patient management since new agents and trial results are providing new perspectives for treatment of prophylaxis and acute attacks.

## 2. Day 2 of Conference: KKS in Disease

Edward Feener from KalVista presented an overview of treatments for HAE based on targets within the KKS. FXIIa mediates the activation of the KKS, which contributes to attacks in HAE. FXIIa inhibitors reduce HAE attacks and may provide future opportunities as antithrombotic agents for a variety of conditions, including cardiopulmonary bypass, catheter-associated thrombosis, LVADS, and other indwelling artificial devices. Multiple PKa inhibitors, including lanadelumab, berotralstat, and ecallantide, are approved for HAE treatment [12]. Additional investigative compounds, including donidalorsen and sebetralstat, are currently in phase 3 trials for HAE treatment. In HAE prophylaxis, the use of donidalorsen, garadacimab, lanadelumab, or berotralstat produced a 90%, 90%, 87%, and 44% reduction in HAE attacks, respectively. The B2R antagonist icatibant is also approved for the on-demand treatment of HAE attacks. On-going studies are examining PKa as a target in diabetic macular edema, including phase 2 studies with KVD001 and THR-149.

Professor Julio Scharfstein from the Federal University of Rio de Janeiro, who is the winner of the Kinin Medal in 2022 for a lifetime of outstanding work in the kinin field, presented his investigations on KKS activation in the host/parasite relationship in Chagas disease [13,14]. *T. cruzi* infection has both an acute and a chronic phase, the latter leading to progressive cardiomyopathy, megaesophagus, and megacolon. In Chagas disease, KKS activation supports the observed acute inflammation, invasion, and local edema by the parasite.

Alessandro Pinheiro of Case Western Reserve University in Cleveland, OH, presented research from the Schmaier and James Kazura laboratories demonstrating that kininogen influences or is influenced by human and mouse cerebral malaria. A total of 42% of plasmas from children with cerebral malaria versus 18% of plasmas from patients with uncomplicated malaria had circulating cleaved HK (cHK) at the time of hospital presentation. Plasma HK levels were also reduced in these children. In a murine model of cerebral malaria [experimental cerebral malaria (ECM)], *Plasmodium berghei* ANKA infection (PbA), *Kng1^−/−^* mice (HK KO) were significantly protected from neurological behavioral deterioration and had a 58% reduction in brain edema based on the Miles assay. Furthermore, PbA-infected *Klkb1^−/−^* mice (PK KO) were also significantly protected from neurological deterioration and had a 64% reduction in cerebral edema. In contrast, PbA-infected *F12^−/−^* mice (FXII KO) were not protected from neurological behavioral deterioration and only had a 33% reduction in cerebral edema. These results indicate that the KKS is activated in human and murine cerebral malaria and deficiencies in HK, PK, and, to a much lesser extent, FXII protect mice from lethal experimental cerebral malaria.

Israel Junior Borges do Nascimento from the Federal University of Minas Gerais presented research that examined liver tissues in patients with SARS-CoV-2 infection. Higher serum *des*-Arg^9^BK and lower BK values were observed in the liver tissues from patients with SARS-CoV-2 infection. Dr. Denis Vincent from the Université de Montpellier, France, presented his observations on the involvement of the KKS in COVID-19-induced lung disease. Lung hyperinflammation may be the result of BK overproduction/accumulation [15].

Luana Sella Motta Maia from the University of Basel presented her studies on a novel homozygous variant in *SERPING1* that has a different interaction with C1s protease and PKa [16]. Two sisters from consanguineous parents have a homozygous missense variant in exon 6 of *SERPING1* (NM_000062.3: c.964G>A; p. Val322Met), which has given them severe HAE since adolescence. Fourteen family members (including the sisters’ parents) are heterozygous carriers of the variant without presenting any symptoms. The Val322Met variant affects a highly conserved position among serpins (80%) that is located within the breach/gate region, thereby inhibiting C1INH from forming a stable complex with C1s protease. This variant makes it impossible to develop the serpin trap due to an alteration in its contact sites.

Marc Vanhove from Oxyuron presented research using THR-149, a bicyclic peptide inhibitor of PKa, in diabetic macular edema that is in a Phase 2 trial. The pharmacokinetics of intravitreous (IVT-PK) and intravenous (IV-PK) administrations were studied [17]. It was found that THR-149 had ocular and systemic half-lives of 36 h and 1.1 h, respectively. It was mostly cleared by the kidneys.

Jeffrey Breit from Rezolute described a novel PKa inhibitor, RZ402, targeted for DME. It has a *K_i_* of 124 nM for PKa and an IC_50_ of 194 nM for BK formation. Its T_1/2_ is 20 h with a peak dose at about 4 h after oral administration. The peak depends on the amount of agent given. In completed phase 1 studies, subjects received oral dosing from 25 to 500 mg for up to 14 days.

Nivetha Murugesan from KalVista presented information on a novel oral FXIIa inhibitor (KV998086). FXII zymogen has intrinsic activity, albeit at 1/4000 of that of FXIIa. KV998086 inhibits FXIIa with an IC_50_ < 10 nM. Following dextran sulfate activation of human plasma, the level of PKa and FXIIa increased 25- and 12-fold, respectively. In the presence of KV998086 > 100 nM, there was nearly complete protection of FXII and PK activation. KV998086 also suppressed FXII zymogen-mediated KKS activation.

Lastly, Antonio Henrique Martins of the University of Puerto Rico examined the molecular mechanisms of the blood–brain barrier (BBB) disruption by a B2R agonist, NG291 [18]. NG291 disrupted the BBB by changing the paracellular and transcellular BBB pathways. NG291 treatment reduced claudin-1 expression and NO formation from one hour to three days, suggesting a paracellular mechanism.

## 3. Conclusions

The KININ 2022 meeting in Annecy presented a wide array of current investigations in the field of FXII, PK, C1INH, and BK, indicating that this field is developing new research contents and venues that are being actively translated to manage diseases.
